# Incidental Findings on CT Scans in the Emergency Department

**DOI:** 10.1155/2011/624847

**Published:** 2011-05-29

**Authors:** Ryan J. Thompson, Susan M. Wojcik, William D. Grant, Paul Y. Ko

**Affiliations:** Department of Emergency Medicine, SUNY Upstate Medical University, Syracuse, NY 13202, USA

## Abstract

*Objectives*. Incidental findings on computed tomography (CT) scans are common. We sought to examine rates of findings and disclosure among discharged patients who received a CT scan in the ED. *Methods*. Retrospective chart review (Aug-Oct 2009) of 600 patients age 18 and older discharged home from an urban Level 1 trauma center. CT reports were used to identify incidental findings and discharge paperwork was used to determine whether the patient was informed of these findings. *Results*. There were 682 CT scans among 600 patients: 199 Abdomen & Pelvis, 405 Head, and 78 Thorax. A total of 348 incidental findings were documented in 228/682 (33.4%) of the scans, of which 34 (9.8%) were reported to patients in discharge paperwork. Patients with 1 incidental finding were less likely to receive disclosure than patients with 2 or more (*P* = .010). Patients age <60 were less likely to have incidental findings (*P* < .001). There was no significant disclosure or incidental finding difference by gender. *Conclusions*. While previous research suggests that CT incidental findings are often benign, reporting to patients is recommended but this is rarely happening.

## 1. Introduction

Computed tomography (CT) scans are commonly used diagnostic tools in the emergency department (ED). It is currently estimated that there are 62 million CT scans done per year in the United States [1]. Commonly included in radiology specialist's interpretation of these scans are findings unrelated to the chief complaint and not pertinent to the immediate patient care in the emergency department. These are classified as “incidental findings.” 

The increased availability and use of CT scans in the emergency department has been well documented [2]. Additionally, the advances in technology of the CT scanners available in most institutions have also increased the resolutions and abilities of radiologists in picking up many more subtle findings. While some of these incidental findings are benign and require no followup, others require serial imaging and close supervision of the patient by their primary care physician [3]. Prior studies have examined rates of incidental findings in trauma patients, a group that receives large amounts of CT scans in their workup [4–7], and renal colic patients [8]. Rates of incidental findings varied from 34% to 43% on abdominal CT scans in trauma patients [4–6], and up to 45% in renal colic ED patients [8]. Rates of proper documentation and referral for followup of incidental findings in these groups has varied from 21% to 27% [7, 8].

No study has ever examined the rates of incidental findings across all CT types in a general ED patient population. Our goal was to explore incidental finding rates in patients who received CT scans in the ED for any reason and were subsequently discharged home and also examine how frequently incidental findings were being disclosed to patients in the discharge paperwork from the ED.

## 2. Methods

Institutional Review Board exemption was obtained for this retrospective chart review. A list of all head, chest, and abdomen/Pelvis CT scans done in the ED at an urban, level 1 trauma center over a three-month period (August 2009 through October 2009) was obtained. This included a total of 2513 patients who had CT scans and were discharged from the ED. These patients were anonymized by putting their medical record numbers into numerical order. Patients were then screened as to their disposition. Only patients who were discharged home following a CT scan were included. 600 patient CT imaging reports and discharge summaries were reviewed.

The reviewer examined the chief complaint as listed in the EMR and, if necessary, the dictated note to determine if a finding was relevant to that patient's current visit and complaints. Then using a structured data collection form, demographic information such as age and gender was recorded along with body area or areas scanned. Finalized CT reports as read by an attending radiologist were used to identify findings. The electronic medical record (EMR) was used to identify a presenting chief complaint for each patient. Then the CT findings were compared to the chief complaints. 

Any findings on CT scan that were not related to the chief complaint were considered “incidental findings.” In all patients with incidental findings the electronic discharge instruction sheet from the EMR was used to assess for disclosure of incidental findings to patients. All evaluation of finalized CT scan reports, electronic discharge paperwork, and determination of “incidental findings” was done by a single reviewer in order to maintain consistency.

Rates of incidental findings were calculated for each body area, as were rates of disclosure to patients. Statistical comparisons and analysis were made for age and gender across the different CT studies performed. A cutoff age of 60 was determined a priori for statistical evaluation. SPSS (Statistical Package for the Social Sciences version 18, SPSS, Inc. now IBM-SPSS) was used for all statistical calculations. Descriptive statistics for categorical and continuous values were computed with alpha <0.05 established for acceptance of statistical significance of differences.

## 3. Results

**Figure 1:**
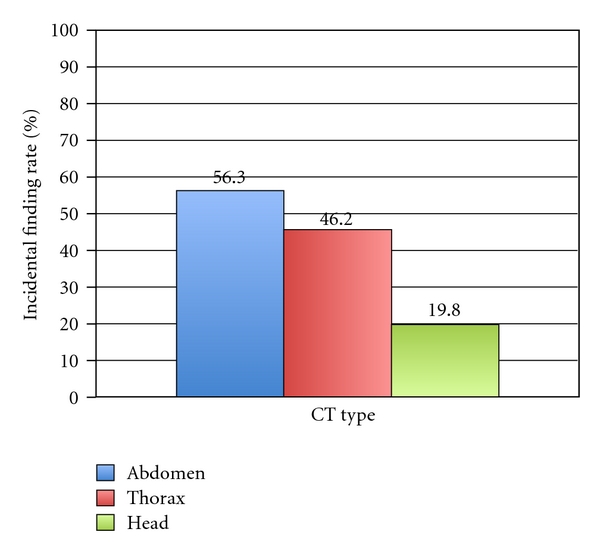
Incidental finding rates by CT type.

There were 682 scans performed on the 600 patients: 405 head CTs, 78 thorax CTs, and 199 abdomen/pelvis CTs (Table 1). At least one incidental finding was identified in 228 scans (33.4%). Abdomen/pelvis scans had the highest rate of incidental findings at 56.3% (Figure 1). There were a total of 348 incidental findings identified using finalized CT reports. Of these, 34 were disclosed to patients in the discharge paperwork (9.8%).  Table 2 demonstrates some of the most common incidental findings and the reporting rates for each. The highest reporting rates were for aortic dilations (33.3%), meningiomas (25%), pulmonary nodules (25%), bony changes (25%), and enlarged adnexa (21.4%). 

Patients with only one incidental finding were less likely to receive disclosure in discharge paperwork than patients with two or more findings (0.08% versus 19.7%, *P* = .01). As expected, patients older than 60 years were more likely to have incidental findings than patients less than 60 years of age (32.5% versus 37.9%, *P* < .001). No significant difference was noted between genders in either rates of incidental findings or rates of disclosure.

## 4. Discussion

Our finding of a rate of incidental findings of 33.4% is similar to that found in previous studies [4, 5]. However, the rate we observed in abdominal CT scans (56.3%) was higher even than the highest rate previously reported (45%) [8]. A prior study examining incidental findings on head CT scans found only a 1% incidental finding rate [9], but that study only considered intracranial findings, whereas our study included extracranial findings such as sinus and bone abnormalities, which may account for the difference. Another possibility is that there may be an increase in the identification and recording of incidental findings by radiologists for medical-legal reasons.

The rate of disclosure of incidental findings to patients via discharge paperwork in this study (9.8%) was considerably lower than the 21% and 27% reported in prior studies [7, 8]. This may be a reflection of the difference between being discharged from the emergency department and being discharged from the hospital as an inpatient, when there is more time to workup these incidental findings and provide appropriate followups. Additionally we did not differentiate between the more concerning incidental findings and the seemingly less concerning findings. We could hypothesize that our reporting rates would have been higher with the more clinically significant incidental findings, although other studies have shown that not to be necessarily true [7]. 

An additional limitation of our study is that, while finalized CT reports were used to identify incidental findings, ED physicians often have to rely on preliminary reports which may not include all these incidental findings. As there is currently no way in our institution to retrieve the preliminary findings in a chart review, we were unable to use the actual reports that the emergency physician had available at the time of discharge. However, it should be noted that, in our facility, preliminary reads are made by senior radiology house staff, and usually reviewed and finalized by an attending radiologist within an hour. As a result, in the majority of cases, incidental findings are being reported on the preliminary read, and, even in cases when they are not, final reports are available within a short time with minimal changes by the attending radiologists.

At our facility, we have a “followup” system in place in which any significant changes, including incidental findings from the preliminary read to the final read, are called from the radiologist to the attending emergency physician, who is then responsible for contacting the patient if they are no longer in the department. However, due to the completeness of preliminary reads at our facility, this is not a common event. In the opinion of the authors, the failure of this study to account for this secondary reporting therefore did not have a significant effect on the results. 

Another limitation is that we utilized only the discharge paperwork that was given to the patient and did not consult the dictated physician note from the emergency department visit. It is possible that some physicians may have given their patients oral instructions regarding any incidental findings. However, the purpose of the discharge paperwork is to give the patient written instructions on how to appropriately followup on their visit, and so the discharge paperwork should reflect all instructions given to the patient. Oral instructions that were not further recorded in the discharge paperwork also represent a failure to fully disclose findings to patients. It is the goal of our emergency department to provide written notice of incidental findings, not merely verbal notice. Also, it is the opinion of the authors that it is within the patient's right to be informed of even seemingly insignificant incidental findings in writing for medical-legal reasons. 

Another possible reason for the low reporting rate is that ED physicians do not consider the findings severe enough to warrant reporting to the patient for followup. However, this cannot be considered a reliable measure of the severity of incidental findings. In a 2008 study of trauma patients by Munk et al., findings were classified into one of three categories depending on severity [7]. The most concerning findings were grouped into Class 3. This included potentially life-threatening findings such as abdominal aortic aneurysm, bone metastasis, ventriculoperitoneal shunt malfunction, esophageal mass, and lung mass consistent with malignancy. Of the 44 class three findings in that study, only 18 (40.9%) had documented followup. Another study by Messersmith et al. examined incidental findings in renal colic patients [8]. The patients were also broken down into three categories based upon the severity of the findings. Of the 61 patients in the “moderate” or “severe” categories, only 11 (18%) had followup of their findings in two years of chart review. 

While our study did not categorize the severity of the findings reported to patients, we did record what the findings were. Some of the most common incidental findings and their reporting rates can be seen in Table 2. While many of these findings may not warrant followup, some represent potentially life-threatening conditions, such as aortic dilations and pulmonary nodules, which only had 33.3% and 25% reporting rates, repectively. Several findings have well-documented guidelines for followup [3]. 

Messersmith's study did show that out of the 11 patients in whom incidental findings had further workup, none had a serious diagnosis [8]. It is therefore important for emergency physicians that inform patients of incidental findings found on imaging to also provide adequate information and counseling as to not alarm patients more than necessary. One author has suggested the term “VOMIT: Victims of Modern Imaging Technology” to describe this phenomenon of the increased number of incidental findings and worry among patients due to the advances in technology and information [10]. Therefore it is imperative of emergency Physicians to inform appropriately these incidental findings as to not overly alarm patients beyond getting appropriate followups with their primary providers. 

Nevertheless, it is apparent that currently we are not doing an adequate job presenting information on the presence and followup of incidental findings to patients. This could result in progression of diseases which could have been treated at earlier stages if proper followup had occurred or a delay in diagnosis of certain diseases. What is needed is a convenient method of informing patients of common incidental findings and their proper followup. Ekeh et al., have proposed the use of a form letter to inform patients when incidental findings are reported after discharge [5]. Other possible ideas include avenues to directly inform a patient's primary care physician of such findings for followup purposes. The addition of prewritten discharge instructions with proper followup for common incidental findings to automated discharge paperwork computer systems could also facilitate emergency physician disclosure of findings to patients. However, a study examining the efficacy of interventions to improve reporting rates is needed.

## Figures and Tables

**Table 1:** Incidental findings and reporting rates by CT type.

CT type	Number of CTs	Rate of CTs with incidental findings	Number of unique incidental findings	Incidental findings reported	Reporting rate
Head	405	19.8%	96	8	8.3%
Thorax	78	46.2%	50	3	8.0%
Abdomen/pelvis	199	56.3%	202	23	11.4%

Total	682	33.4%	348	34	9.8%

**Table 2:** Reporting rates for most common incidental findings.

Incidental finding	Numbers found	Numbers reported	Reporting rate (%)
*Head*			
Meningioma	4	1	25.0
Sinus changes	31	3	9.7
*Thorax*			
Pulmonary nodule	16	4	25.0
*Abdomen/pelvis*			
Aortic dilation	3	1	33.3
Bony changes	12	3	25.0
Enlarged adnexa/adnexal cyst	14	3	21.4
Liver lesion	27	4	14.8
Abdominal wall hernia	9	1	11.1
Nonobstructing renal calculi	10	1	10.0
Hiatal hernia	16	0	0.0
Renal lesion	11	0	0.0
Diverticula	10	0	0.0
Cholelithiasis	6	0	0.0
